# A rare case of cardiac myxoma with light bulb–like cystic morphology: a case report

**DOI:** 10.1093/ehjcr/ytad331

**Published:** 2023-07-21

**Authors:** Shutaro Futami, Michinari Hieda, Mitsuhiro Fukata, Akira Shiose

**Affiliations:** Department of Hematology, Oncology and Cardiovascular Medicine, Kyushu University Hospital, Clinical Research Building B6F, 3-1-1 Maidashi Higashi-ku, Fukuoka, Japan; Department of Hematology, Oncology and Cardiovascular Medicine, Kyushu University Hospital, Clinical Research Building B6F, 3-1-1 Maidashi Higashi-ku, Fukuoka, Japan; Department of Hematology, Oncology and Cardiovascular Medicine, Kyushu University Hospital, Clinical Research Building B6F, 3-1-1 Maidashi Higashi-ku, Fukuoka, Japan; Department of Cardiovascular Surgery, Kyushu University Hospital, Fukuoka, Japan

**Keywords:** Cardiac tumour, Myxoma, Cystic tumour, Feeding artery, TEE, Coronary angiography, Case report

## Abstract

**Background:**

Cystic myxomas are quite rare. Moreover, few reports have evaluated the causes that constituted them.

**Case summary:**

A 73-year-old Asian man presented for pre-operative examination of osteoarthritis, and transthoracic echocardiography (TTE) revealed an incidental intracardiac mass. Therefore, he was referred to our department for further evaluation. He had no specific symptoms or family history related to tumours and heart failure. The TTE showed a 32 × 24 mm spherical mass adherent to the left atrial septum. The upper part of the mass was cystic in formation and hypoechoic inside and resembled a light bulb. Transoesophageal echocardiography showed the feeding arteries flowing from the bottom into the cystic part. In addition, two jet strips drained from the cystic part in the direction of the mitral valve. Coronary angiography revealed the feeding arteries, which consisted mainly of the right coronary artery conus branch and the left circumflex branch, and the blood flowed into the saccular area from the feeding arteries and excreted towards the mitral valve. Surgical resection was performed due to the mobility, and the histopathology confirmed a cystic myxoma.

**Discussion:**

We described the unique anatomical formation of a cystic myxoma, which consisted of an exquisite balance between the tumour-feeding arteries and the draining outlet vessels.

**Learning points**

- To evaluate a differential diagnosis of cardiac tumours from their site of origin, morphology, tumour markers, other laboratory findings, and multimodality imaging studies.
- To consider the origins of the tumour morphology from the detailed evaluation of the images.

## Introduction

Primary cardiac tumours are a rare group of diseases^1^ and sometimes have unexpected morphology.^2,3^ Among cardiac tumours, a cardiac myxoma occasionally contains cysts, necrotic tissue, fibrosis, haemorrhage, or calcification.^4^ Among them, cystic myxomas are rare, with only a few cases documented in the literature.^3,5–7^ Herein, we reported a particularly inimitable form of a cardiac cystic myxoma and its anatomical and physiological conditions.

## Summary figure

**Figure:**
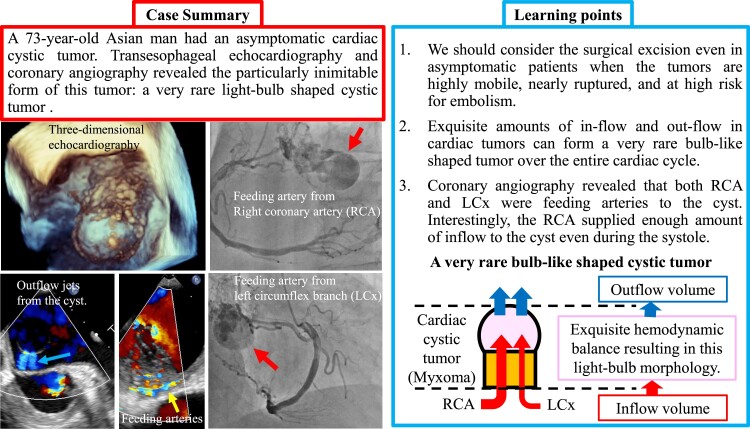


## Case summary

A 73-year-old Asian man suffered from bilateral knee joint pain 4 months ago. Therefore, he visited an orthopaedic surgeon and was diagnosed with osteoarthritis. In addition, transthoracic echocardiography (TTE) for pre-operative screening revealed an intracardiac mass in the left atrium. Therefore, he was referred to our department for further evaluation and treatment.

**Figure 1:**
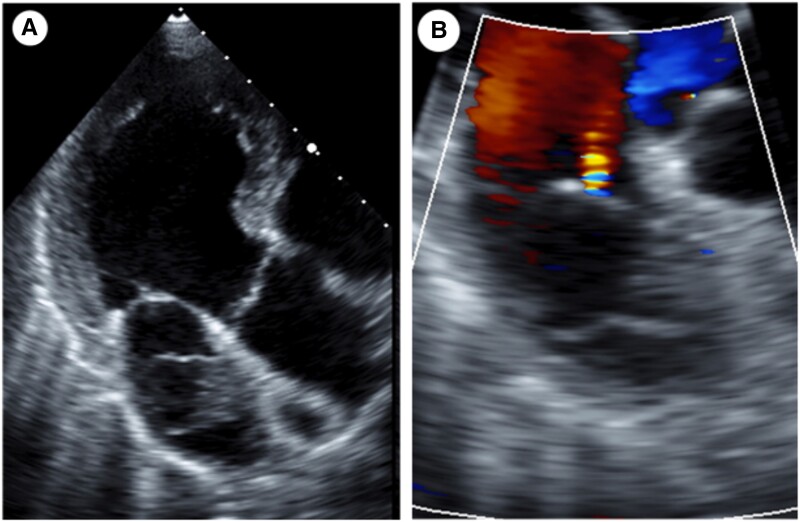
Transthoracic echocardiography showed a mobile tumour attached to the left atrial septum (*A*). A single jet was observed from the cystic lesion (*B*).

**Figure 2:**
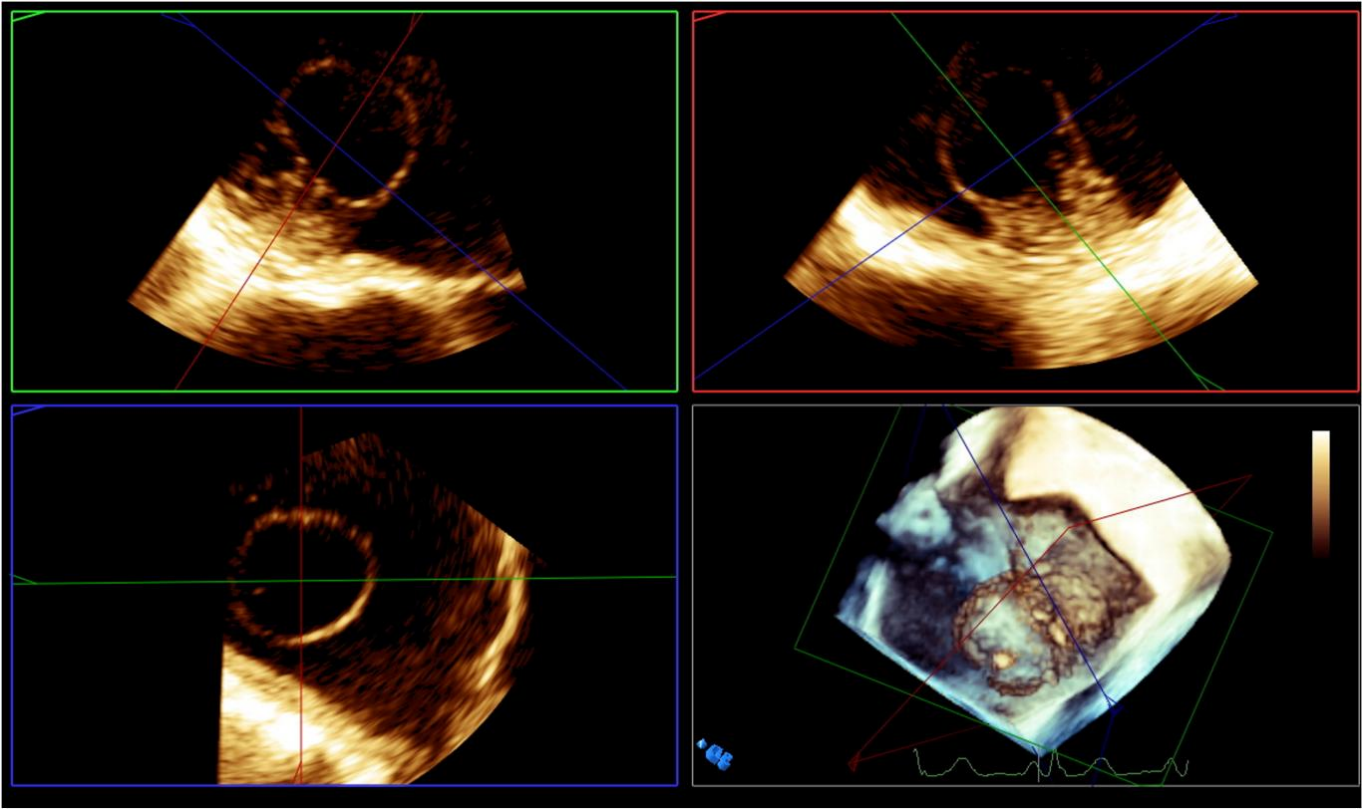
A three-dimensional image of this tumour by transoesophageal echocardiography showed the light bulb–like morphology.

**Figure 3:**
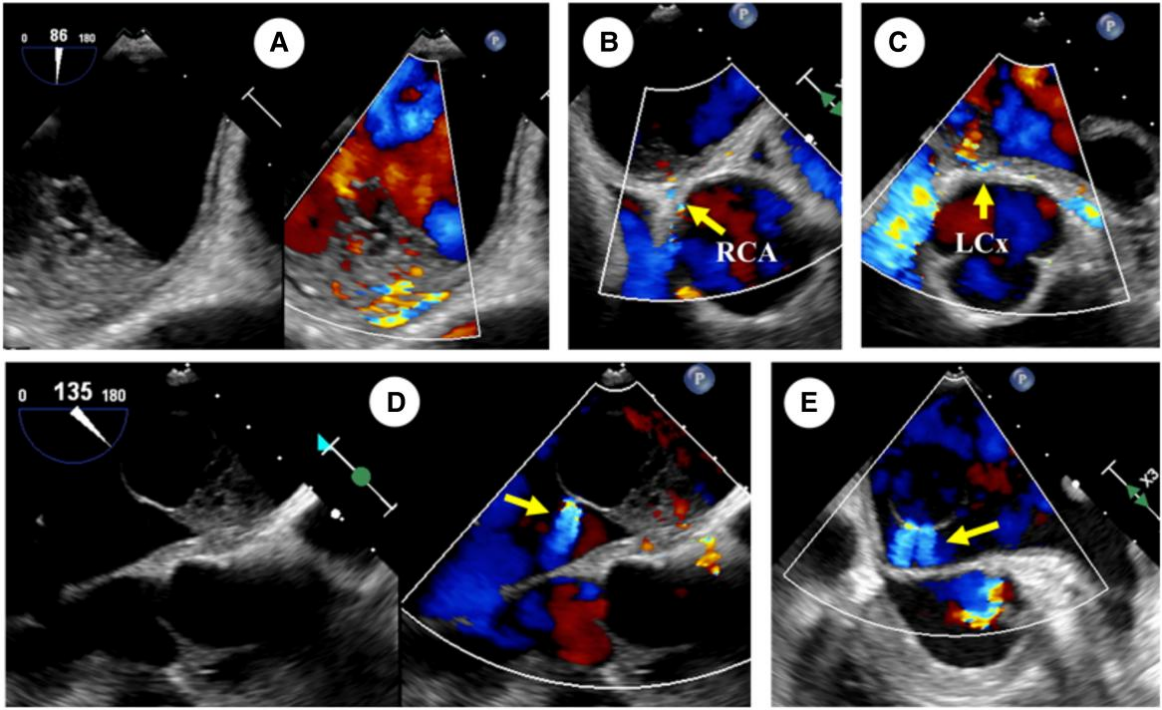
The images of the blood inflow and outflow of the cystic lesion by transoesophageal echocardiography. The Colour Doppler image showed that the feeding arteries flowed from the bottom of the tumour into the cystic lesion (*A*–*C*), and twin jets drained from the cystic part (*D*, *E*). RCA, right coronary artery; LCX, left circumflex branch.

The patient had no medical history or family history related to tumours and heart failure. Previously, he had never had palpitation or dyspnoea. His height and weight were 156.3 cm and 62.2 kg. There were no significant findings in the physical examination, including heart auscultation. Blood tests were within normal limits, including the blood coagulation system, tumour markers, and immunity-related items. His electrocardiogram showed normal sinus rhythm without significant ST-T changes. The TTE showed a 32 × 24 mm mass adherent to the left atrial septum (*Figure 1*; see Supplementary material online, *Video S1*). The base part of this mass was solid and isoechoic, and the upper part was cystic and hypoechoic inside. A jet blew from the cystic mass towards the mitral valve (MV). Transoesophageal echocardiography (TEE) showed a mobile pedunculated tumour like a light bulb (*Figure 2*; see Supplementary material online, *Video S2*). In addition, careful observation revealed the feeding arteries flowing from the bottom into the cyst (*Figure 3A–C*; see Supplementary material online, *Video S3*) and twin jets draining from the cyst towards the MV(*Figure 3D* and *E*; see Supplementary material online, *Video S1*). These jets from the cyst were observed throughout all phases of the cardiac cycle.

**Figure 4:**
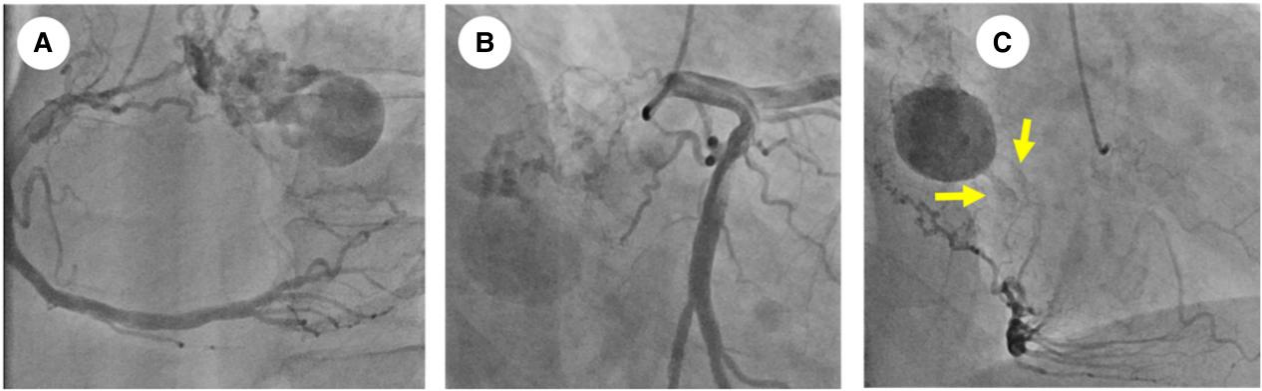
Coronary angiography showed that the feeding arteries consisted of the right coronary artery conus branch (*A*) and the left circumflex branch (*B*). Twin jets from the tumour were observed (*C*).

**Figure 5:**
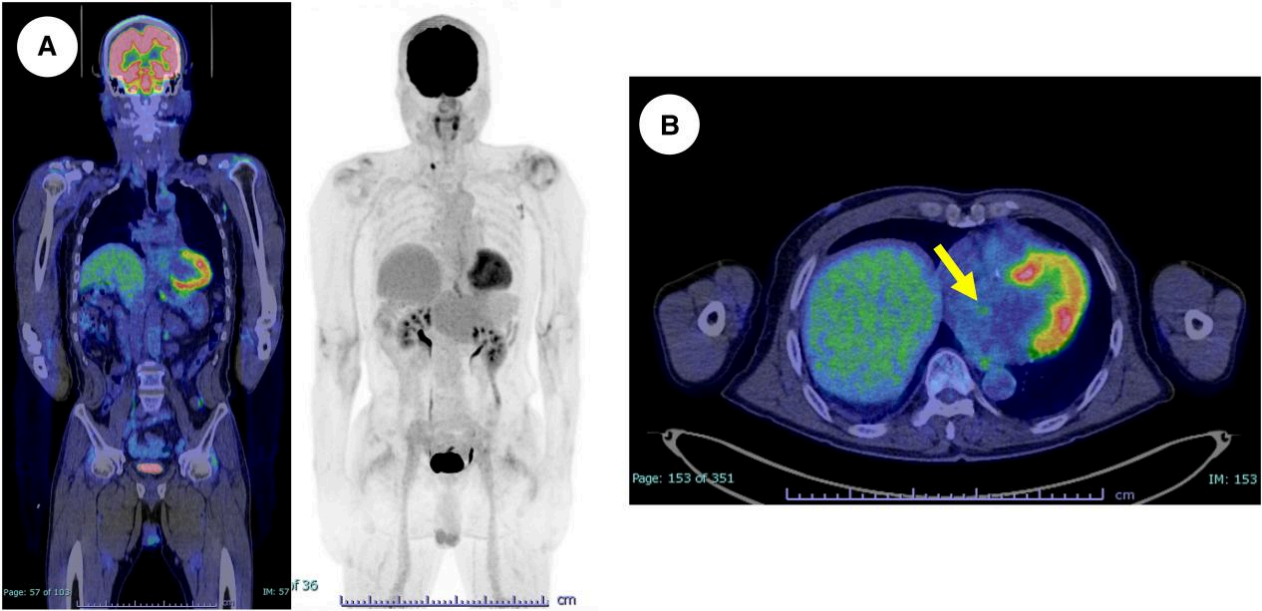
The fluorodeoxyglucose–positron emission tomography indicated no metastasis in other organs (*A*). The left atrial tumour had mild accumulation (the max standardized uptake value: 2.4) (*B*).

Coronary angiography (CAG) revealed the feeding arteries, which consisted of the right coronary artery (RCA) conus (*Figure 4A*; see Supplementary material online, *Video S2*) and the left circumflex branch (LCX) (*Figure 4B*; see Supplementary material online, *Video S3*). The blood supply from the feeding arteries was relatively dominant from the RCA. The CAG also showed blood flow entering the cyst from the feeding arteries and excreting towards the MV. Similar to the TEE findings, the twin outlet jets were confirmed (*Figure 4C*; see Supplementary material online, *Video S4*). The computed tomography and fluorodeoxyglucose–positron emission tomography (FDG–PET) indicated no metastasis in other organs but mild accumulation in the left atrial tumour (the max standardized uptake value: 2.4) (*Figure 5*).

Differential diagnoses included myxoma, lipoma, haemangioma, undifferentiated pleomorphic sarcoma, angiosarcoma, or metastatic tumours. We ruled out metastatic cardiac tumours because (i) the tumour markers were within normal range, and (ii) there was no abnormal accumulation other than this cardiac tumour on FDG–PET. In addition, considering the morphology of the cystic mass, the site of origin, and the frequency of cardiogenic tumours, we suspected a benign cardiogenic tumour, especially a cystic myxoma.

Though this patient was asymptomatic, he was at very high risk for embolism due to the nearly ruptured form of the tumour. We explained the potentially life-threatening situation. After informed consent, we performed the surgical excision. The base of the tumour was centred in the fossa ovalis and was extensively attached to the atrial septum on the left atrial side. The base component was solid, and the apical portion was cystic. In addition, we confirmed several little feeding arteries to the cyst during tumour resection.

Histopathologically, the section revealed a proliferation of ovale-shaped cells with slightly hyperchromatic nuclei arranged in a cord-like pattern embedded in an abundant myxoid matrix (see Supplementary material online, *Figure S1*). These features were compatible with a cardiac myxoma. His postoperative status was stable. He lived without any symptoms, and there was no recurrence of cardiac tumours on TTE in October 2022.

## Discussion

Cardiac tumours have the risk of embolization from the cardiac tumour itself or thrombi formed on its surface. We performed the surgical excision even in this asymptomatic patient because the present tumour was highly mobile, nearly ruptured, and at high risk for embolism.

The present case was a particularly unique form of a cardiac cystic myxoma, and three rare conditions were necessary for this formation: (i) the feeding arteries and amount of blood supply into the cyst, (ii) the adequate size of the drainage holes and outlet flow volume from the holes, and (iii) the physical strength of the cystic membrane of the cardiac myxoma. In particular, (i) and (ii) were essential.

The TEE revealed the feeding arteries to the cyst, which caused this cystic formation in the myxoma. Furthermore, the interesting point was that no collapse of the cyst occurred throughout all phases of the cardiac cycle. If the inflow to the cyst continued to exceed the outflow, the cyst would enlarge and eventually rupture. Conversely, if the amount of inflow was less than the outflow, the cyst would be compressed by left atrial pressure and collapse. In this case, neither of these events occurred. The echocardiography showed that the cyst remained approximately the same size throughout all phases of the cardiac cycle. These findings suggested that the positive inner pressure of the cystic myxoma was always maintained even or higher than left atrial pressure.

Interestingly, the feeding arteries consisted of the RCA and LCX, and the RCA was relatively dominant. Generally, the left coronary artery (LCA) flow is biphasic in the cardiac cycle and prevalent during the diastole. In contrast, the RCA flow is approximately equal during the systole and diastole due to its property of supplying to the right heart system.^8,9^ Therefore, if the feeding arteries only consisted of the LCA, the cyst would not obtain adequate inflow during the systole, resulting in collapse. In addition, the remarkable amount of blood flow from the feeding arteries to the cystic myxoma might have resulted in a coronary steal phenomenon and typical anginal symptoms. Coincidentally, there were no symptoms related to ischaemic heart disease in this case. Thus, these feeding arteries, including the RCA, made it possible to enter an adequate amount of blood into the cyst in both volume and time. Furthermore, this inflow and the outlet flow volume from the twin holes of the cyst made an exquisite haemodynamic balance, resulting in this extremely rare light bulb tumour morphology.

## Conclusion

This case was a cardiac myxoma with a unique light bulb morphology in the left atrium. The delicate balance between the inflow and the outflow of the cystic myxoma kept the positive internal pressure of the cyst over cardiac cycles, resulting in the unusual formation of this light bulb morphology.

## Supplementary Material

ytad331_Supplementary_Data

## Data Availability

The data underlying this article are available in the article and in its online Supplementary material.

## Lead author biography

Dr Shutaro Futami graduated in 2012 at Kyushu University, Fukuoka, Japan. He is currently working as a cardiologist at Kyushu University Hospital, Fukuoka, Japan. His main areas of interest are echocardiography, valvular diseases, heart failure, and geriatrics.
