# Supplementary material for: A rare case of cardiac myxoma with light bulb–like cystic morphology: a case report
Source: Eur Heart J Case Rep. 2023 Jul 21;7(8):ytad331. doi: 10.1093/ehjcr/ytad331 (PMC10398420; doi:10.1093/ehjcr/ytad331)
Supplement: ytad331_Supplementary_Data [file ytad331_supplementary_data.zip › Light bulb Timeline_submit_revised2_cleanr_ver.docx]

**Timeline**

| **Date** | **Events** |
| --- | --- |
| 7^th^ January 2020 | A 73-year-old Asian man visited an orthopedic surgeon and was diagnosed with osteoarthritis. A transthoracic echocardiography (TTE) for preoperative screening revealed an intracardiac mass in the left atrium. |
| 27^th^ January 2020 – 2^nd^ February 2020 | He was referred to our department for further evaluation and treatment. TTE showed a 32 x 24 mm spherical mass adherent to the left atrial septum. The upper part of the mass was cystic in formation with hypoechoic inside and resembled a light bulb. |
| 26^th^ February 2020 | The surgical excision was performed for a definitive diagnosis and to prevent sequelae like shower-embolic multiple strokes or angina pectoris. The histopathology confirmed cystic myxoma. |
| 25^th^ October 2022 | The patient lived without any symptoms, and there were no specific findings in TTE suspected recurrence of cardiac tumors. |
