# Supplementary material for: A rare case of cardiac myxoma with light bulb–like cystic morphology: a case report
Source: Eur Heart J Case Rep. 2023 Jul 21;7(8):ytad331. doi: 10.1093/ehjcr/ytad331 (PMC10398420; doi:10.1093/ehjcr/ytad331)
Supplement: ytad331_Supplementary_Data [file ytad331_supplementary_data.zip › Supplemental Appendix_(revised)_clean_ver.docx]

**Supplemental material**

**Figure S1. Histopathological images**

Histopathologically, the section revealed a proliferation of ovale-shaped cells with slightly hyperchromatic nuclei arranged in a cord-like pattern embedded in an abundant myxoid matrix. (Hematoxylin and eosin staining, A:40x, B:200x)

**Supplemental Video S1. The images of the twin jets from the cystic lesion by transesophageal echocardiography**

The color doppler image of transesophageal echocardiography showed the twin jets excreted toward the mitral valve during all cardiac phases.

**Supplemental Video 2. Coronary angiography (Right coronary artery)**

The coronary angiography showed the feeding artery from the right coronary artery conus branch.

**Supplemental Video 3. Coronary angiography (Left coronary artery)**

The coronary angiography showed the feeding artery from the left circumflex branch.

**Supplemental Video 4. Coronary angiography (twin jets from the cystic lesion)**

The coronary angiography showed that the blood flowed into the cystic lesion from the feeding arteries and excreted toward the mitral valve during all cardiac phases.
