# Supplementary figures and images for: A rare case of cardiac myxoma with light bulb–like cystic morphology: a case report
Source: Eur Heart J Case Rep. 2023 Jul 21;7(8):ytad331. doi: 10.1093/ehjcr/ytad331 (PMC10398420; doi:10.1093/ehjcr/ytad331)

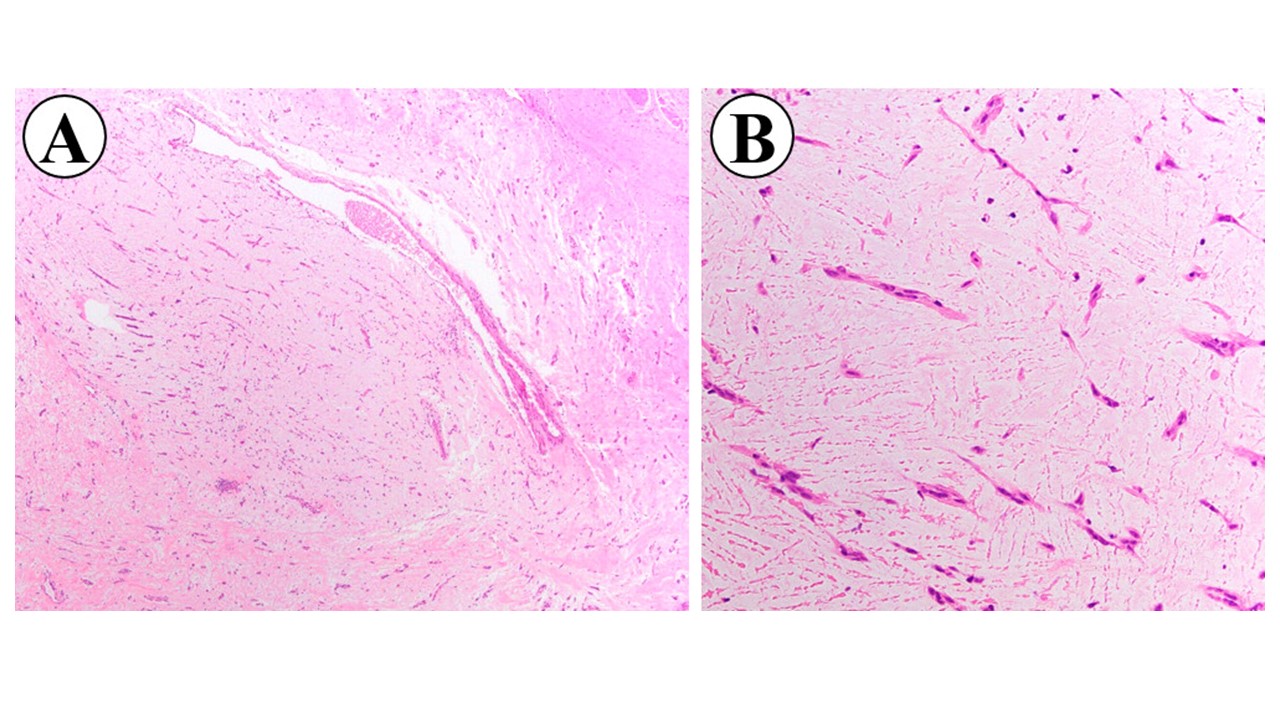

Supplement: ytad331_Supplementary_Data [file ytad331_supplementary_data.zip › Figure S1.jpg]
